# A general procedure to identify indicators for evaluation and monitoring of nature-based solution projects

**DOI:** 10.1007/s13280-022-01740-0

**Published:** 2022-05-31

**Authors:** Anne Rödl, Alessandro Arlati

**Affiliations:** 1 Institute of Environmental Technology and Energy Economics, Hamburg University of Technology, Eißendorfer Strasse 40, 21073 Hamburg, Germany; 2 Department of Urban Planning and Regional Development, HafenCity University Hamburg, Henning-Voscherau-Platz 1, 20457 Hamburg, Germany

**Keywords:** Co-creation, Decision-making, Evaluation, Indicator selection, Monitoring, Nature-based solutions

## Abstract

In the last years, the idea of using natural elements or nature-based solutions (NbS) to mitigate the impacts of cities on climate, biodiversity and citizens' health became more popular in research and practice. Nevertheless, there are currently uncertainties in finding and selecting appropriate criteria and indicators for monitoring and evaluating the impact and performance of NbS and its co-creation processes. This paper proposes an easy-to-use and structured procedure for selecting appropriate criteria and indicators for monitoring and evaluating any kind of NbS project. The user is guided step by step in selecting meaningful metrics. The procedure is tested using a real case study from the Horizon 2020 research project CLEVER Cities as an example. The test shows that by following the indicated procedure, the criteria and indicator selection process is speeded up and reproducible.

**Supplementary Information:**

The online version contains supplementary material available at 10.1007/s13280-022-01740-0.

## Background and objective

The impacts that urbanization has caused in the last decades on global and local climate, ecosystems and human health are well known (Brown and Otto 2021). Among others, land-use change and uncontrolled land sealing are often accompanied by loss of natural habitats and soils (Seto et al. 2011). As these kinds of challenges increase in number and intensity (IPCC 2019), multidisciplinary strategies built upon systemic approaches that foresee the collaboration among different types of stakeholders at all levels of government, i.e. multi-level governance model (see for instance Benz et al. 2007), to protect water supplies, address habitat loss and mitigate and adapt to climate change in cities are needed (Reside et al. 2018). Since the 2010s, there has been a growing awareness of the properties of nature to provide ecosystem services as cost-effective and flexible alternatives in urban policy and spatial planning to respond to these challenges (Groot et al. 2010; European Commission 2013; Gómez-Baggethun and Barton 2013; Geneletti et al. 2020). For some years now, the term 'nature-based solutions' (NbS) as a type of ecosystem services has gained popularity (see Ronchi et al. 2020). The term NbS was first used in 2008 in the context of various ecosystem services that support biodiversity and climate change adaption by a World Bank publication (The World Bank 2008; Potschin et al. 2016). In the current understanding, one of the aims of NbS is to reconnect the built environment with more nature-driven systems, bringing nature back into the cities (European Commission 2015; Kabisch et al. 2017).

There have been several attempts to define the term “nature-based solutions”. The European Commission defines the term 'nature-based solutions' in their research and innovation agenda on nature-based solutions (European Commission 2015) as:

“Actions that are inspired by, supported by or copied from nature” that “help societies to address a variety of environmental, social and economic challenges in sustainable ways.”

According to Cohen-Shacham et al. (2019), NbS is an umbrella concept covering a whole range of ecosystem-related approaches. Cohen-Shacham et al. (2019) distinguish five categories: restorative, issue specific, infrastructural, managerial and protective ecosystem-related approaches. NbS relate to the utilization of ecosystem services or the natural capital for human purposes and are often found in urban planning or civil engineering practices (Science for Environment Policy 2021). In addition, the commonly agreed definition from the International Union for Conservation of Nature (IUCN 2020) emphasizes the idea of using ecosystem services for human welfare.

As visible from these different definitions, NbS can refer to different types of interventions, ranging from the management of protected natural areas to street trees or retention ponds (see Table 1 for more examples). There are many requirements and expectations regarding their benefits for human well-being and biodiversity (Science for Environment Policy 2021) but also fears of unintended consequences like green gentrification or socio-spatial inequalities (e.g. Haase et al. 2017).

**Table 1:** Definitions of key terms used in this article

**Evaluation** • Denotes an act of judging the amount, quality, value or importance of something (Cambridge Dictionary 2014b) • Checking results of an intervention in a specific moment • Quality, value or significance judgments are derived by comparing the data to reference values—i.e. the effectiveness of the output is observed by comparing the results with the desired objectives	
**Monitoring** • Watching and observing a situation carefully over a period of time • Serves the collection of data and does not include an act of judgement (Cambridge Dictionary 2014d) • Refers to an ongoing process, where, for example, the progress of a project's evolution is checked • Actions of longer duration can be monitored • Monitoring of series of activities in time that stop after achieving their objectives (e.g. co-creation actions) is not possible	
**Criteria** • Standard by which you judge, decide about or deal with something (Cambridge Dictionary 2014a) • In this article, we define the term “criterion” as a distinguishing feature or characteristic of a system, product or process that is considered essential and can be used to judge or compare different systems, products or processes • Expressed in the form of the desired requirement or outcome, usually related to a specific topic	
**Indicators** • Carrier of information describing an element (Haase et al. 2014) that helps to reflect a complex task that is not measurable by itself (Dusseldorp 2014) • Are based on available data with quantitative or a qualitative measure • It specifies a “criterion” in more detail; one criterion can be specified by more than one indicator • Should be clear, traceable and adequately reflect the facts of the case (Dusseldorp 2014) • They must be measurable, easy to handle and valid for different regions (Gudmundsson et al. 2010) • “**Key performance indicators (KPIs)**” are often used in business administration to measure the performance or progress of an organization, project or product (Cambridge Dictionary 2014c) • KPI selection depends on what is deemed necessary for the individual case and requires a good understanding of the situation under evaluation and its objectives	
**Reference** • Reference values provide a benchmark to which the measured indicator values can be related • Chosen carefully according to the selected criteria and indicators of the specific case	

However, developing and implementing NbS concepts can be challenging and complex (Eggermont et al. 2015; Cohen-Shacham et al. 2016; Bush and Doyon 2019; Babí Almenar et al. 2021), especially if co-creative approaches are deployed to include and consider diverse stakeholders and their interests (Frantzeskaki 2019). Besides a lack of basic knowledge and capacity in municipal planning (Wamsler et al. 2017), a lack of financial resources (Droste et al. 2017), or the lack of a regulatory framework specifically for NbS (Wamsler et al. 2020), there is a need to prove the effectiveness of nature-based interventions (Chausson et al. 2020) and to provide guidance and advice for replication (Sowińska-Świerkosz and García 2021). Evaluation and monitoring of the co-creation processes and the impacts of NbS projects play a supportive role to gain experience and facilitate future NbS design decisions (Kabisch et al. 2016; Braubach et al. 2017; Wamsler et al. 2017; Mahmoud and Morello 2021; Sowińska-Świerkosz and García 2021). Evaluation and monitoring of established NbS constructions are key to maximize benefits and to avoid negative impacts or the repetition of mistakes in the future projects (Xing et al. 2017). Therefore, the selection of appropriate and meaningful criteria and indicators is crucial for the informative value of the assessment results (Dumitru and Wendling 2021; Science for Environment Policy 2021; Sowińska-Świerkosz and García 2021).

However, the complexity of NbS projects, i.e. various NbS types, implementation scales, stakeholder interests, conflicting goals, working with natural processes, lack of data, etc., makes it challenging to select appropriate assessment criteria and indicators, as the variables are numerous, and case-dependent (Sowińska-Świerkosz and García 2021). Also Dumitru and Wendling (2021) report on the challenges of finding appropriate indicators for assessing NbS projects and point out that a sound scientific method should be included in the process of (co-)defining indicators. However, a simple, structured and reproducible procedure for selecting appropriate criteria and indicators for the evaluation and monitoring of NbS is still not explored sufficiently. Therefore, this article proposes a procedure that can potentially be applicable for the selection of appropriate criteria and indicators for the assessment of any type of NbS at different phases of its co-creation pathway. The procedure can be used to find appropriate criteria and indicators for the ex-post assessment of NbS co-creation processes, its results or the development of ex-ante assessments. The proposed procedure is designed to ease the evaluation and monitoring of NbS as evidence-based supporting tool.

## Definition of terms

When dealing with assessment, there is often confusion between the terms evaluation and monitoring within the literature. Hence, the two terms are used in this paper according to the definitions presented in Table 1. Within this article, we intend the term ‘assessment’ as an overarching term comprising both evaluation and monitoring.

Similarly, the terms “criterion” and “indicator” are often used very differently in the literature. Therefore, definitions of these terms are also presented in Table 1.

## Existing approaches for NbS evaluation and monitoring

There are several overarching NbS assessment frameworks existing so far. In the following, we shortly describe some of them because they can be used as a basis for our step-by-step approach for criteria and indicator determination, presented later in Section “Structure of the proposed criteria and indicator selection approach”. For example, Raymond et al. (2017a) and Kabisch et al. (2016) suggested NbS assessment frameworks, while other authors provided an overview of criteria and indicators (IUCN 2020).

The EKLIPSE approach (Raymond et al. 2017a) is one of the most cited frameworks. It consists of a holistic framework that identifies how NbS provide ecosystem services and socio-economic benefits in urban areas. It is designed to be applicable during various stages of NbS projects. In this sense, Raymond et al. (2017a) and Raymond et al. (2017b) can be used as a reference repository for suitable indicators for NbS impact assessment. A recent publication by a joint task force built of a large group of experts from EU H2020 NbS projects (Dumitru and Wendling 2021) developed further the ten challenges from Raymond et al. (2017b) into 12 separate societal challenge areas. They further specified the Raymond et al. (2017a) framework with tools to plan and indicators and methods to assess NbS.

Besides knowledge gaps and barriers related to NbS, Kabisch et al. (2016) identified indicators for assessing the effectiveness of NbS. A further framework attempt has been presented by Watkin et al. (2019). The framework consists of 5 steps and aims to quantify the benefits and co-benefits of implemented NbS including guidelines for selecting indicators.

Also some EU Horizon 2020 and INTERREG projects have developed collections and knowledge bases on assessment methods for urban NbS (NATURVATION 2021), monitoring technologies (Somarakis et al. 2019) or a set of measurement and data collection methods for monitoring and comparison of NbS (Wendling et al. 2019). Huthoff et al. (2018) presented a framework for evaluating the added value of NbS compared to grey solutions. They also propose four groups of indicators and provide some guidance how the indicator groups can be related to NbS and their assessment.

To date, there are only a few other approaches that explicitly address the process step of selecting an appropriate set of criteria and indicators for NbS assessment. Sowińska-Świerkosz and García (2021) provide a procedure guided by performance questions to receive a set of indicators for selecting effective NbS projects. They explicitly refer to the selection phase of the NbS co-creation pathway (see phase 2 in Section “Phases of NbS co-creation and assessment needs”) and not to the project evaluation phase. Also Dumitru and Wendling (2021) integrated an approach for developing monitoring and evaluation strategies including the selection of suitable indicators.

So far, the literature scan on existing approaches to support NbS assessment revealed a rather broad yet discordant panorama of frameworks that try to support the complex task of indicator finding, as also argued in Calliari et al. (2019). Nevertheless, especially the rather broad frameworks and attempts do not provide the practitioner with concrete guidance on how to identify and select appropriate criteria and indicators for his or her individual project. In fact, other than stating that indicators should be selected for the assessment, they do not elaborate on how exactly or what selection criteria might be used to accomplish this task. On the other hand, it is obvious that it would not be possible for any outside author to provide a complete set of indicators for every individual type of NbS project a priori. Therefore, in the next section, we do not propose another assessment framework but provide a structured and reproducible approach for selecting an individually tailored set of criteria and indicators for an NbS project, taking into account the plethora of assessment frameworks already available in literature.

## Phases of NbS co-creation and assessment needs

**Fig. 1:**
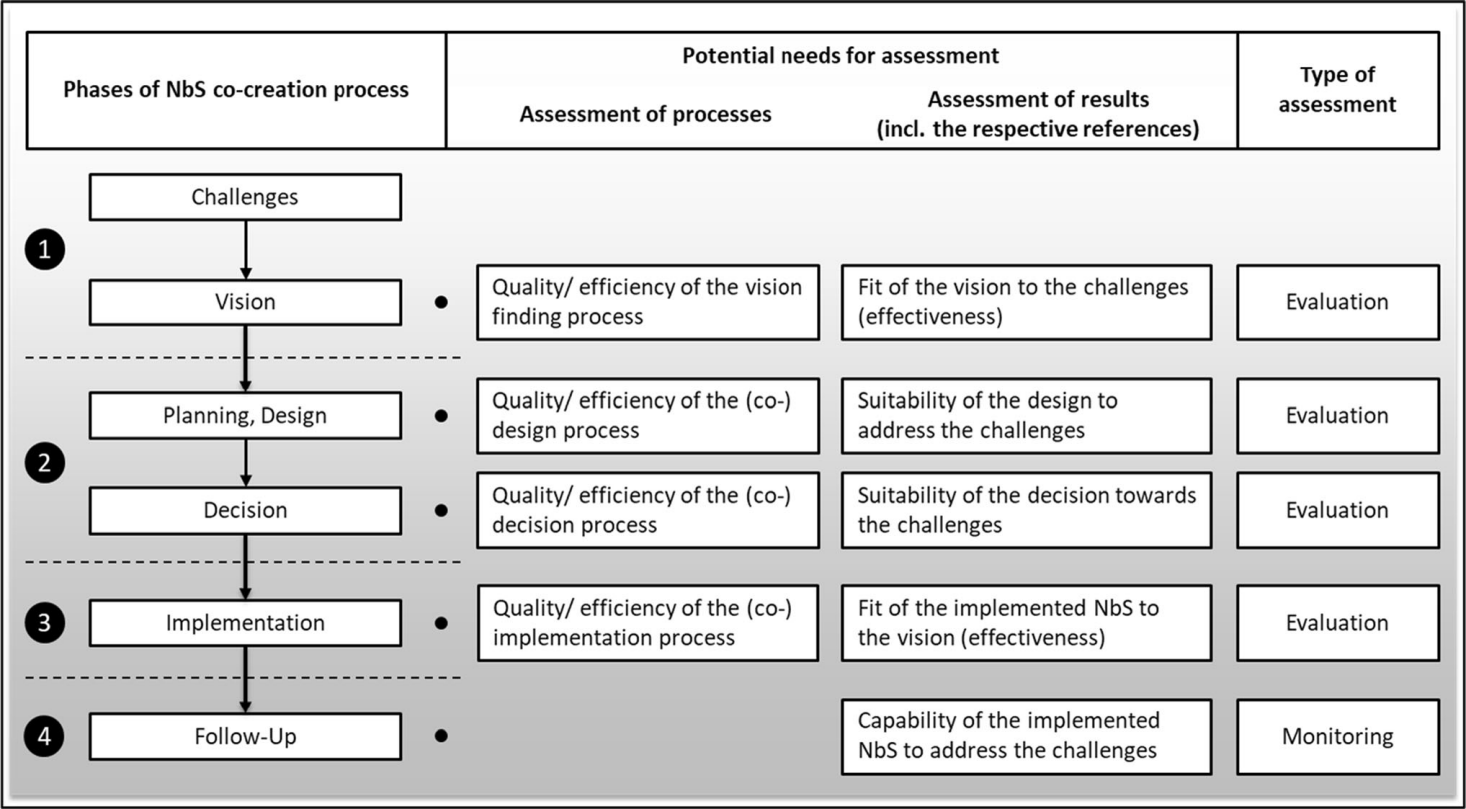
Potential assessment needs in the different phases of NbS co-creation processes (own figure)

The approach chosen by the European Commission for NbS implementation envisages a co-creative process that requires the involvement of various stakeholders. Assessment of the effectiveness of the involved processes and the impacts of the final interventions could be required in all of the phases (Fig. 1). Therefore, the NbS co-creation process phases are described in more detail below.

According to the scheme for NbS implementation proposed by Raymond et al. (2017b) and other more recent works like Morello et al. (2018) or Albert et al. (2020), we define four major phases of the NbS co-creation process:

1. *Challenges and visions definition*
  The first phase of an NbS project concerns defining the key challenges that the intervention will address and developing a vision of what the outcome should look like. How a challenge is understood is critical, as this will inevitably influence the direction of the planning and implementation process and shape the NbS intervention. Local stakeholders can be involved in the process as a repository of local knowledge.
2. *NbS design and decision*
  In the design stage, the focus is on developing possible NbS intervention types. In the decision stage, the most appropriate actions to address the challenges and vision are selected. Design and decisions processes can be made jointly with residents and other local stakeholders.
3. *NbS implementation*
  Implementation involves building or setting up the NbS. In this phase, the output of the NbS co-creation process materializes and becomes visible. The result is usually something that can be touched, seen, heard, or smelled. NbS implementation can involve residents or be done in collaboration with other stakeholders.
4. *Follow-up*
  The final phase aims to assess the impact of the implemented NbS to rate their quality or effectiveness related to the addressed challenges. This monitoring process can take years after the completion of the NbS co-creation process. Depending on the monitoring results, the NbS can be replicated or scaled up.

Each stage within the phases of a NbS co-creation process (Fig. 1) has different assessment needs. The quality or efficiency of processes or the results of these processes can be the subject of the assessment. As also shown in Fig. 1, in most cases, the assessment type will be an evaluation. As defined in Table 1, monitoring is only possible when a NbS is completed, and its development can be observed over time. That is why monitoring usually takes place in the follow-up phase.

Each phase of the NbS co-creation process has different objectives to consider when selecting criteria and indicators.

## Structure of the proposed criteria and indicator selection approach

As previously argued, determining suitable criteria and indicators is challenging since these are characteristic of the individual project scope and its phases. Therefore, the here-proposed approach will not give recommendations for specific criteria and indicators but rather will provide a structured step-by-step procedure that allows the user to select appropriate criteria and indicators for his or her particular purpose. The procedure is designed to be adaptable to any type of NbS project. It formalizes the identification of appropriate criteria and indicators for monitoring and evaluation of all relevant NbS co-creation phases.

**Fig. 2:**
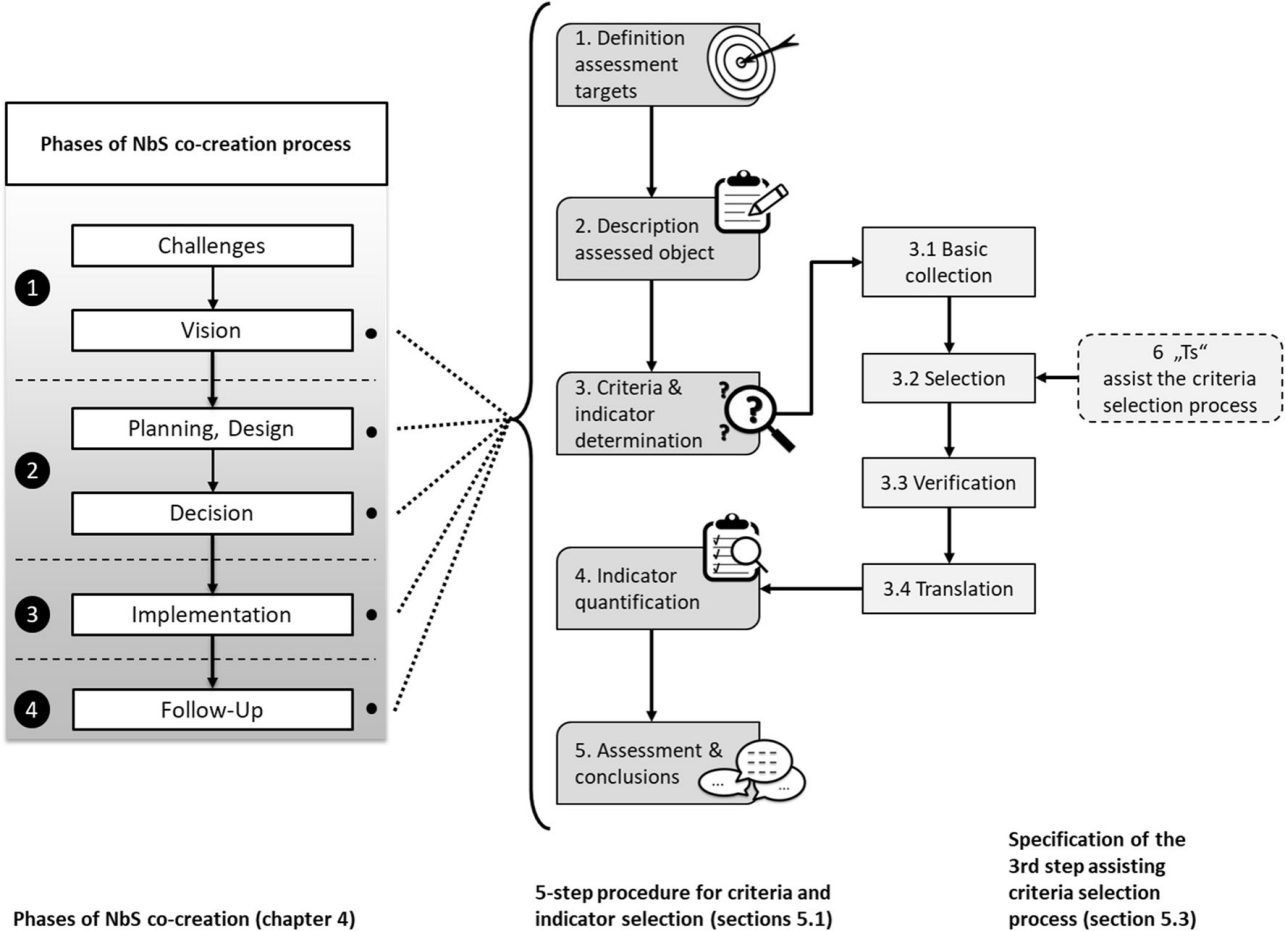
Overview of the entire criteria and indicator selection approach described in this section (own figure)

The starting point for the development of the process was the challenge of identifying appropriate indicators for the assessment of the NbS interventions to be implemented in the EU-H2020 CLEVER Cities project (CLEVER Cities 2021). At that time, there was little concrete guidance and experience with the actual indicator finding. After screening the literature, the authors brainstormed on how the indicator search process could be structured, standardized, and thus facilitated. The procedure was elaborated and discussed in front of other project partners as well, which provided important feedback for its refinement. The resulting procedure logically structures the complex information that must be considered in the assessment of NbS. Each step is translated into targeted questions that automatically guide the user to the appropriate criteria and indicators. In the following, Fig. 2 provides an overview of the entire concept. The individual elements are then explained in more detail in the next sections.

The proposed 5-step procedure for criteria and indicator selection consists of four core steps and one step that involves feedback to the NbS planning and development process, namely:

1. Definition of assessment targets
2. Description of the assessed object
3. Determination of suitable criteria and indicators
4. Data collection to quantify the indicators
5. Assessment and conclusions to provide feedback to NbS planning, design or implementation

**Fig. 3:**
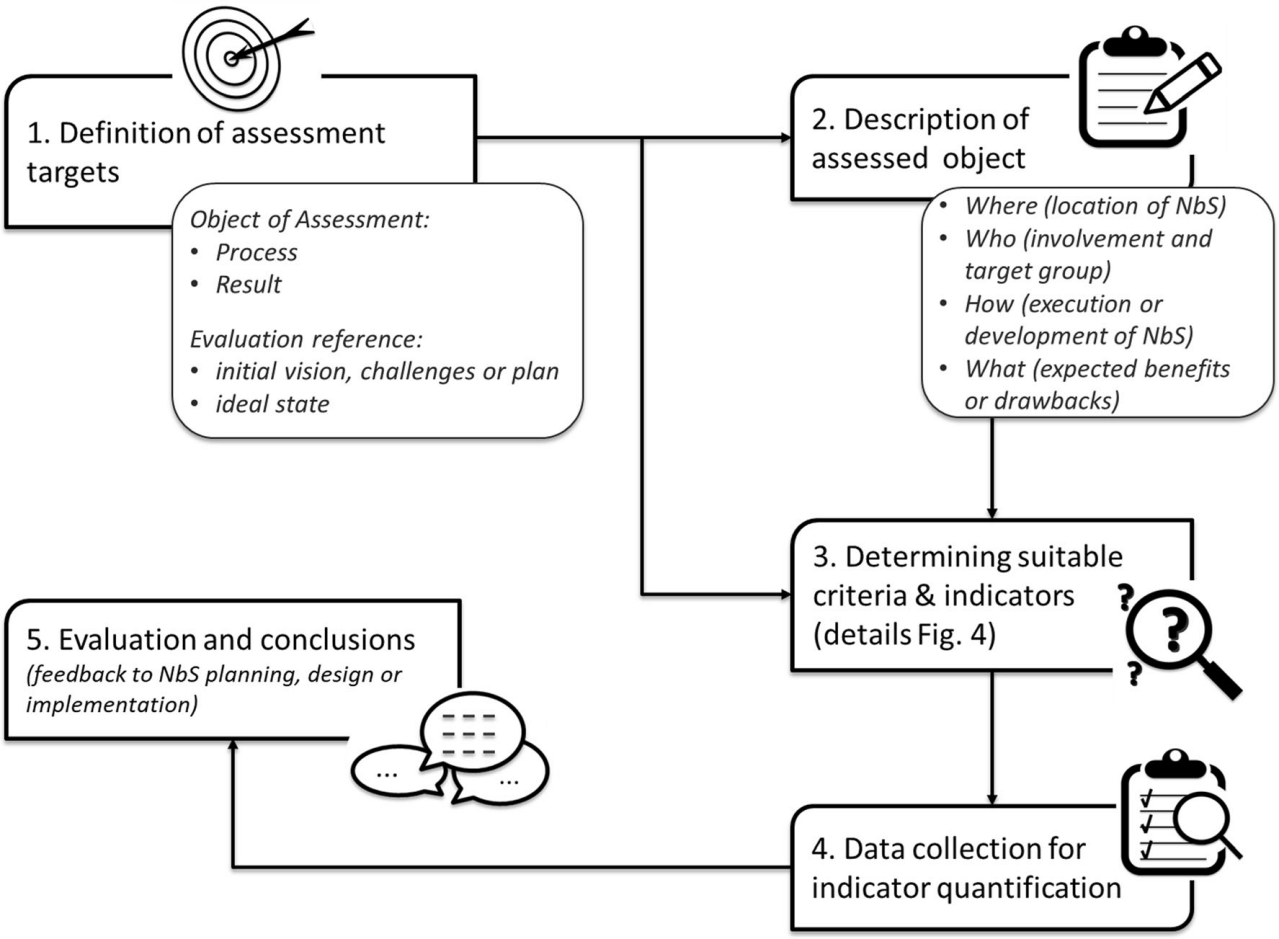
Five-Step procedure for finding appropriate criteria and indicators (own figure)

The procedure can be applied in each of the phases of the NbS development process mentioned above (see Fig. 1) whenever criteria and indicators are needed for evaluation or monitoring. Figure 3 provides an overview of the 5-step procedure described in the following Sections (“Step 1: Definition of the assessment targets”–“Step 5: evaluation and conclusions”). Before starting, the definition of a set of stakeholders to engage in the criteria and indicators finding is crucial as well. Depending on the type of the project, there should be always a mix between stakeholders type, following the quadruple helix principle (Carayannis and Campbell 2009) and the co-creation approach. In particular, the presence of a local agency for the engagement of citizens, experts on NbS-related aspects and interest groups should be guaranteed (Arlati et al. 2021).

### Step 1: Definition of the assessment targets

The most crucial step is to define the purpose of the assessment. The person performing the assessment should clarify together with all interested parties what the desired information from the assessment should be. The 1st step includes the description of the scope and the selection of the assessment object, i.e. whether the assessment is aimed at a process of NbS co-creation or its outputs (see Fig. 1). In addition, thought should be given to the required level of detail of the assessment. At this point, it is advisable to recapitulate the challenges that led to the initiation of the NbS co-creation process. What was the initial situation? What were the challenges to be solved? These work should usually have already been done in phase 1 of the NbS co-creation process (Fig. 1). The information received from this step should describe the overall targets of the NbS project to get an idea of the initial intention. By making this clear, it will be easier to state the specific goal of the assessment. For example, if a community garden is built to improve the social cohesion in the neighbourhood, different indicators are needed than if the intent is to increase biodiversity in the district.

A suitable reference should also be selected or defined if an evaluation is planned, i.e. the baseline or target against which the results are compared (see Table 1).

### Step 2: Description of the assessed object

After defining the assessment objective, the assessed object, i.e. the co-creation process or output, has to be described in detail. The questions below will help to structure the description:

- Where is the NbS built, where the development process takes place?
  - *Describe here the location as detailed as possible – this might include information about the climate, geographical information and structure (buildings, social, green spaces)*
- Who is involved, and who is affected from the process or the NbS implementation?
  - *Here, the different types of stakeholders should be considered and analyzed, particularly participants of the NbS project and other affected persons.*
- How is the NbS implemented or the process executed?
  - *Describe the methods that have been used to implement the NbS or to steer the process. This is especially important if co-creative processes are included in the assessment.*
- What benefits or potential drawbacks are resulting from the implemented NbS or the process executed?
  - *Describe the expected benefits and potential drawbacks arising from the implementation of the NbS. The description can refer to the fulfillment of the societal challenges described by Raymond et al. *(2017a)* and the Dumitru and Wendling *(2021).

### Step 3: Determining suitable criteria and indicators

**Fig. 4:**
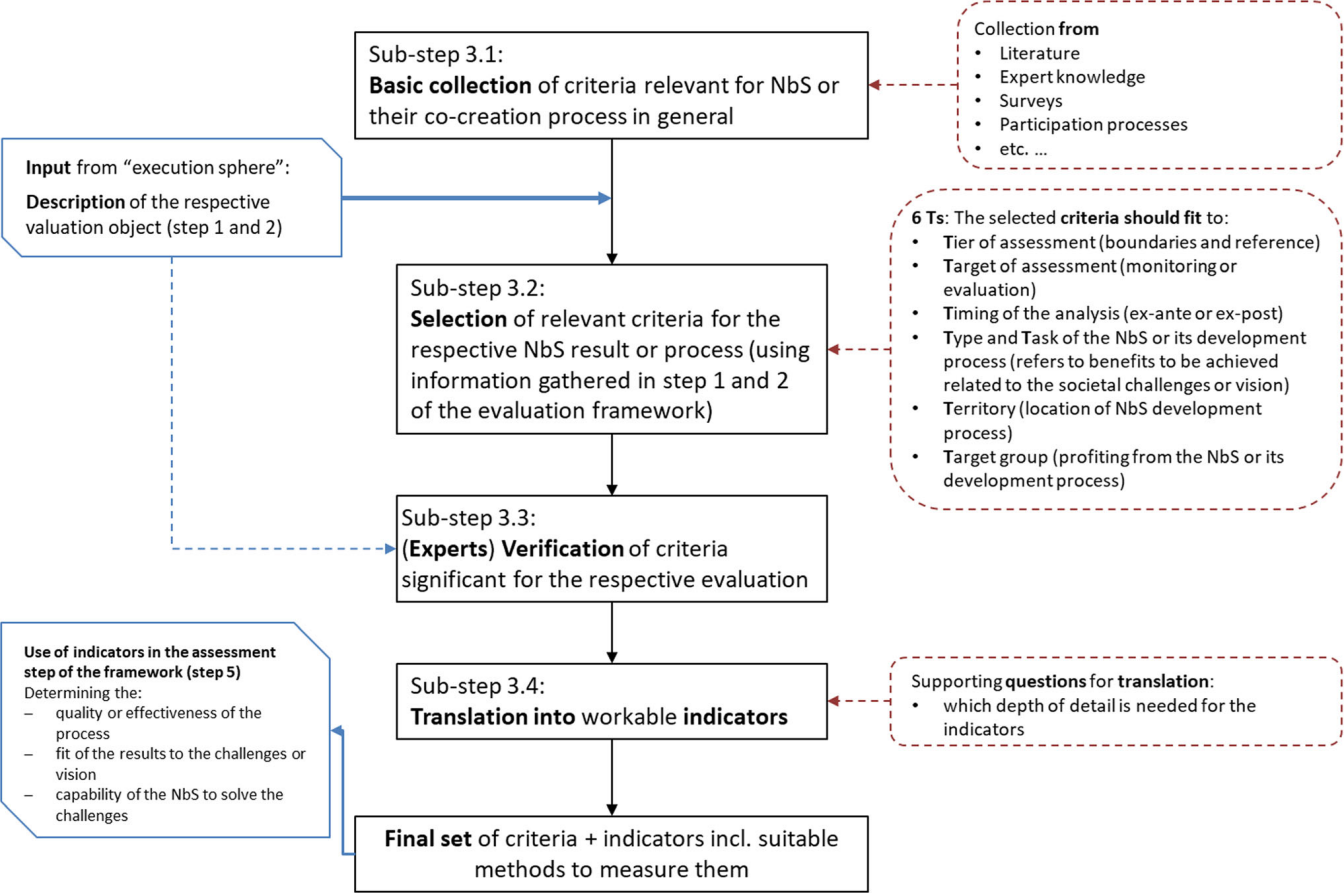
Route for criteria and indicator selection as part of step 3 of the proposed procedure (own figure)

After all, elements have been described and understood in the previous steps, the criteria and the corresponding indicators can be selected. To do this, Step 3 of the procedure provides detailed instructions. As mentioned earlier, criteria are distinguishing features or characteristics of a system, product or process that are considered essential (Table 1). They must first be selected to set the frame for the assessment. Figure 4 provides an overview of the sub-steps leading to the selection of appropriate criteria and their indicators. Especially in substep 3.2, some guiding principles are offered that can ease the selection process.

The criteria selection process begins with a basic collection of criteria relevant to characterizing NbS services or the success of their co-creation process (substep 3.1). There are already various approaches and guidance documents that provide a range of topics for NbS assessment from which potentially suitable criteria can be compiled. See, e.g. Wendling et al. (2019) or Raymond et al. (2017b), Calliari et al. (2019), IUCN (2020), NATURVATION (2021), Dumitru and Wendling (2021), Sowińska-Świerkosz and García (2021), Watkin et al. (2019) and further more (see Section “Existing approaches for NbS evaluation and monitoring”).

In substep 3.2, the following six “Ts” are provided to support the selection of appropriate criteria from the basic collection:

- **T**ier of assessment (boundaries and reference)

This first “T” refers to the boundaries of the assessment. They need to be defined in terms of their **spatial dimension**, e.g. which areas are included in the assessment and which are not, and whether the assessment is done at the macro-, meso- or micro-level. Furthermore, the **temporal dimension** of the evaluation or monitoring needs to be determined. What is the monitoring timeframe, e.g. how many years after implementation should the development be observed? Also, when evaluating a process, the timing of data collection needs to be determined, e.g. for surveys, when people will be interviewed or how often (before, during or after the start of the co-creation process).

- **T**arget of analysis (points level, evaluation or monitoring)

The point of assessment, according to Figure 1, needs to be clarified. This second 'T' corresponds to the question “what” in step 2 and clarifies **whether** an **evaluation or** a **monitoring** (or a combination of the two) is aimed at and **whether** the **process or** the **result** should be analysed within the respective phase. Appropriate indicators should be selected concerning the specific target of analysis*.* For example, for monitoring purposes, it would be advisable to choose practicable indicators, i.e. those that can be easily collected with available sensors, methods or tools, even in the long-term period. The same applies to indicators for process evaluation, even if the analysis is only done once and not continuously.

- **T**iming of the analysis (ex-ante or ex-post)

There is a difference whether the analysis is done before the implementation of a NbS (ex-ante) or whether the NbS output or process result is analysed (ex-post). An ex-ante analysis would provide the opportunity to adjust the plans if shortcomings are identified. Noteworthy, as a rule, the ex-ante analysis cannot be more than a **look into the future** with incomplete information about the exact course of the NbS implementation process. An ex-post analysis means that the **process is completed** and the result is visible. In most cases, this also means that nothing can be changed. The analysis results can only be used to learn about the quality of the process or the results for future projects or replications.

- **T**ype and **T**ask of the NbS or its co-creation process (description of the object)

These “Ts” correspond to the description of the object under study explained in step 2. The **T**ype of NbS ideally corresponds to the **challenges to be addressed and determines** the characteristics and impacts of the NbS. In most cases, only a few criteria and indicators will fit the particular context in the end. Several publications (e.g. Ramírez-Agudelo et al. 2020; Somarakis et al. 2019) propose a classification of NbS types, primarily based on the typology proposed by Eggermont et al. (2015), which is shown in Table 2.

To determine the ‘**T**ask’, it is essential to identify at least one main objective to fulfill the NbS or its co-creation process. The task refers to the benefits to be achieved in relation to the societal challenges or vision. A list of 10 challenges that NbS can alleviate is found, e.g. in the EKLIPSE framework by Raymond et al.(2017a) or in its latest version from the Dumitru and Wendling (2021).

**Table 2:** Overview of the typology of NbS proposed by Eggermont et al. (2015) and further elaborated by Somarakis et al. (2019)

	NbS Type	Intensity of engineering	Examples of interventions^1^
1	Better use of natural ecosystems	Minimal interventions	Protection of terrestrial or aqueous ecosystems
2	Sustainability and multi-functionality of managed ecosystems	Effective management	Landscape management (e.g. pest or weed management, creation of habitats, erosion control, water resource management)
3	Design & management of new ecosystems	Transformational approach	Urban green spaces (parks, green strips, trees) blue-green spaces establishment (riparian buffers); green built environment (green roofs and facades), natural water storage and infiltration (rain gardens)

^1^ For a complete list of the classification scheme and its subcategories, see Somarakis et al. (2019) and for a non-exhaustive list of NbS intervention examples see Dumitru and Wendling (2021)

- **T**erritory (location of NbS)

This “T” relates to the question “**where**” in step 2. The location correlates with the **scale of implementation**. Here, for example, it can be described whether the NbS is established in a city or the countryside (built environment, other habitats or nature elements), and what the climate, soil conditions, competing flora or fauna are like (e.g. do predators harm the new greenery, do fast-growing species inhibit growth, etc.). These aspects are crucial for the assessment of types 1 and 2 of NbS (Table 2).

- **T**arget group (interested in or potentially benefit from or otherwise affected by NbS)

This last “T” refers to the question “**who**” in step 2, and it is an essential point to consider when looking for appropriate criteria and indicators. NbS are mainly aimed at improving people's situation in many ways. Therefore, it is essential to consider the **target group** of the respective NbS intervention or its implementation process and identify which **social groups are involved** and **who will benefit** from the NbS output or process. These groups are often referred to as stakeholders. A stakeholder who actively participates in decision-making can be called an actor (see Dente 2014). The list of stakeholders is always very specific to the project in question. There are methods for stakeholder identification and analysis (e.g. Dente 2014; Prell et al. 2009; Reed et al. 2009; Kivits 2011; Leventon et al. 2016) or see, e.g. Yang (2014) or Lelea et al. (2014) for an overview of different methods. Nevertheless, there might also be groups that could be adversely affected by an NbS intervention. It would therefore be necessary to identify these groups as well, e.g. in order to think about possible compensatory measures.

The resulting list of the criteria should be critically reflected and verified by experts (substep 3.3).

In the final substep (3.4), the selected criteria are further specified with practicable indicators to operationalize them, i.e. finding measurable parameters that help assessing the respective criterion's status and development*.* When selecting suitable indicators, specific general selection requirements described in Table 1 should be considered (Gudmundsson et al. 2010).

In the Supplementary Material, you can find a table (Table S1) illustrating exemplarily the various types of criteria and indicators suitable for the different phases and assessment objects of NbS co-creation.

### Step 4: Data collection and quantification of indicator values

Once the final list of indicators has been determined during step 3, selecting suitable assessment methods and measurement tools can begin (step 4 in Fig. 3). Suitable tools can be, e.g. surveys, interviews, observations, mobile applications or other digital tools, maps, GIS (geographic information system), simulation programs, sensors, etc.

Most of the time, data collection needs to start before the implementation of an NbS in order to have a reference to compare the development against. The period between the assessment of the status quo and the assessment of the final impacts needs to be carefully defined. It should be noted that different impacts may show up in different periods. Therefore, it should be carefully decided when which data is collected.

### Step 5: evaluation and conclusions

In a final step (5 in Fig. 3), the collected data must be processed, archived and assessed. Conclusions must be drawn from the results obtained. The results help to rate the quality of the NbS co-creation processes or their outputs. With their help, feedback and recommendations for planning, design or implementation process can be derived.

## Application

**Fig. 5:**
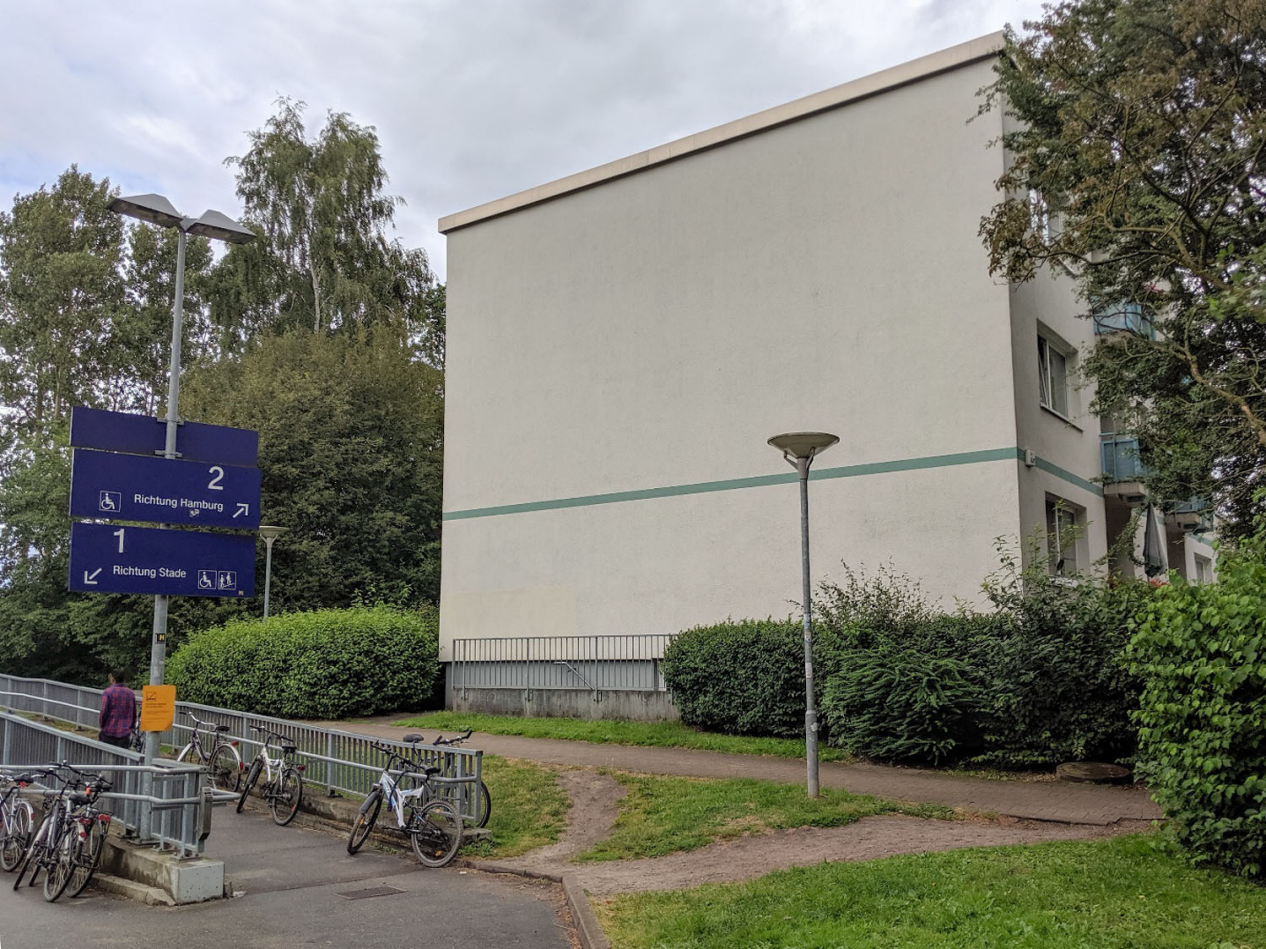
Façade that will be greened in the course of the project (CLEVER Cities 2021) and that was chosen as the subject of the exemplary application of the criteria and indicator finding procedure proposed in this article (own image 2021)

In this section, the procedure presented above is now demonstrated by the authors using a case study from the above-mentioned Horizon 2020 project (CLEVER Cities 2021). The project, running from 2018 to 2023, aims to improve urban neighbourhoods in Hamburg, London and Milan to ameliorate the residents' social cohesion, well-being and security. To illustrate the potential application of the previously described procedure, an example from Hamburg is taken. The chosen example refers to a green façade that will be installed in cooperation with the local housing cooperative at a multistory building in a social housing estate. Figure 5 gives an impression of the current state of the façade. It is located close to a suburban train station, which is why a lot of people pass by every day. The project is currently closing phase 2 of the co-creation process (see Fig. 1). The process that brought us to the final decision to implement a NbS on this building was a combination of different needs and interests (see “Objectives” in Table 3) from the participants of a series of expert workshops (see “Who?” in Table 3). Among others, the Theory of Change (TOC) methodology (see eg. van Es et al. 2015) was used to facilitate the discussion during phase 1 (see Fig. 1). The project is intended as a pilot to gain experience with façade greening and also to show its acceptance and appreciation by the people in a deprived neighbourhood. The question is also whether such a façade can contribute to identification with the place and can create a certain pride for the place. Phase 3 will start in the third quarter of 2022, but, however, already now we would like to select our criteria and indicators for monitoring the implementation results in the follow-up phase (see Fig. 1).

**Table 3:** Compact overview of the individual steps of the method using the example of a green façade (own table)

*Step 1—Definition assessment targets and frame*	
Targets of assessment:	Frame is the EU-Project (CLEVER cities) that addresses among others at social cohesion, place regeneration and knowledge and social capacity building With the help of the facades, following challenge should be addressed: – Façade is barren, not very aesthetic (all houses look the same) → green is missing in a concrete jungle – Few experience with vertical green at residential buildings – Deprived neighbourhood → low participation, expectation and interest from the residents and passers-by Assessment target: – Monitoring of the development of the green façade itself (growth) and the impacts of establishing the façade on the house and the surroundings (environment, people)
Reference (to be compared to)	Façade without green (bare)
*Step 2—Description of the assessed object*	
Where?	Façade at a multistory house in a residential area at the fringe of a big city
Who?	Involved: housing company, gardeners, city administration, residents, urban development agency Profiting: residents, passers-by, housing company, city of Hamburg
How?	Planning: jointly, including residents in choosing plant species Implementation: co-creation (together with residents)
What are the potential positive effects?	– Increasing urban green area – Local biodiversity enhancement – Upgrading of the building and neighbourhood – Increasing Knowledge and Capacity Building for urban green
What are the potential drawbacks?	– Green gentrification – Increase of maintenance costs → Increase of rent prices – Residents are passive users of the façade and do not care about it – Attraction to vandalism
*Step 3—Determining suitable criteria & indicators*	
Basic collection	Long list derived from literature (see Section “Existing approaches for NbS evaluation and monitoring”): Raymond et al. 2017a; Wendling et al. 2019; IUCN 2020; Dumitru and Wendling 2021; NATURVATION 2021
**Reduction of relevant criteria according to:**	
Tier	1. Spatial: building with façade, close surrounding (neighbourhood)—micro-scale 2. Temporal: monitoring five years after implementation, in a yearly rhythm
Target	3. Monitoring of the established façade
Task	4. Ex-post analysis
Territory	5. Residential area in a district in the outer parts of a big city with 1.9 Million inhabitants in Germany, low social and economic conditions
Target group	– Residents of the house, interest in having a better living place, potentially paying reduced energy bills – Residents in the close surrounding, interest in the aesthetic of their living area – Housing company: interest in increasing satisfaction of their tenants, publicity of their project, gaining experience with green façades – Passers-by, interest in aesthetic
*Reduced list of relevant criteria and indicators:*	
Criteria	1 Monitoring of the façade and house development (plant growth, insulation, condition of the built façade, plaster, colour) 2 Satisfaction and gained experience in the housing company 3 Building inhabitants satisfaction 4 Passers-by impression 5 Residents proud of place
Workable indicators (cross-check criteria and addressed challenges)	1 – Area of green reached within monitoring period (1 year) – Area of the façade (colour, plaster) damaged or in a bad condition 2 – Degree of the consent of the housing company with the façade – Number of employees of the housing company with new skills related to green façades 3 – Number of residents (in building/neighbourhood) content with the green façade – Change of degree in satisfaction of the place among residents (in building/neighbourhood) – Perceived temperature in the summer season in the adjacent apartments 4 Number of passers-by expressing consent to the establishment of the façade 5 Change of degree in proud of place among residents (in building/neighbourhood/passers-by)
*Step 4—Data collection for indicator quantification*	
– Surveys/Interviews with residents and passers-by – Measurement of heating demand (comparison before and after) – Visual estimation of façade condition (survey with passers-by) – Measurement of the vegetated area of the façade	
Step 5—Assessment and conclusions* (*hypothetical)	
– Area of the façade is increasing year by year – Residents are content with the façade (especially residents with a view of the façade) – Passers-by like the aesthetics – Change in heating demand is not measurable – Condition of the façade is deteriorating more slowly than on buildings without green façade	

The demonstration of the step-by-step procedure focuses on the steps 1 to 3 (Fig. 3). Steps 4 and 5 are rather practical tasks and dependent on real-life measurements, which have not yet been executed in the project. For step 4, suitable tools for measurement and surveying are proposed. The assessment results in step 5 are purely hypothetical and do not correspond to real project outcomes. In the following central points of the procedure, execution will be explained and its results are displayed in Table 3.

Step 1 of the procedure is quite straight forward. The goal of the assessment has to be clarified. In the example, we are aiming for monitoring the results of our NbS intervention and how the final green façade is fitting to the challenges and needs in the neighbourhood. In our case, there first was the vision to realize such a façade for a social housing building. The expected benefits were derived from examining similar projects in the literature and during the TOC workshop. This façade would be a pilot project for the housing company to develop a solution that could eventually be standardized for other buildings with similar features in the city. The neighbourhood in which the façade is implemented is characterized by economically and socially deprived situation. For instance, language barriers prevented the direct participation of many residents of the neighbourhood. This greatly forced the project team to look for simple and quick alternatives to solicit residents’ opinions on design of the façade and plant selection. Since there is relatively little experience with façade greening in Hamburg, further challenges arose during the planning phase (phase 2 in Fig. 1).

Against this background, the criteria selection later in step 3 will relate to technical issues (e.g. construction materials, gained technical and organizational experience) and will also take particular account of local social conditions (e.g. inhabitants satisfaction, appreciation of passers-by, sense of belonging). In step 3, therefore only criteria and indicators will be preselected that relate to technical or societal issues. Further specification takes place in step 2 by answering the questions where, who, how and what. Later in step 3, further narrowing down of the appropriate criteria is done by answering the 6 “Ts” (Fig. 4) in substep 3.2. All criteria not fitting to the answers in step 2 and 3 will be withdrawn from the basic collection. The final list of criteria has then to be translated into operable (measurable) indicators in substep 3.4. This step is not easy to predefine in advance by outsiders, i.e. in guidelines or manuals.

The translation into feasible indicators is very depending on the individual scale of the project, financial and human resources for monitoring, know how and available technical equipment. Not all indicators that are measurable are also feasible in the individual case. Guidance for determining suitable indicators is provided by the answers in step 2 and substep 3.2. Inspirations and a number of suitable indicators for different criterion topics can be found in the literature mentioned (Section “Existing approaches for NbS evaluation and monitoring”), e.g. in Dumitru and Wendling (2021). In our example, we consulted experts, extracted information from the literature and decided on indicators, making a trade-off between meaningfulness and feasibility.

## Discussion

This article describes a procedure that structures and guides the process of indicator identification in the context of monitoring and evaluation of NbS projects. The procedure guides the user through the process of indicator selection by asking specific questions. Furthermore, the procedure could be used for other purposes where indicators are needed for monitoring and evaluation, e.g. sustainability assessments.

As the exemplary application of the procedure in Section “Conclusions” demonstrated, there are many factors that need to be taken into account when putting the procedure into practice. Since the procedure is intended to be workable for many different purposes within the sphere of NbS assessment (NbS types, phases of the co-creation pathway), it needs individual inputs and decisions to receive appropriate indicators. Compiling a primary list is the responsibility of the user. It has not been shown in detail here. The preliminary list of criteria and indicators includes more or less all criteria found in the literature and elsewhere that fit the purpose and scope of the assessment as constrained by the answers to the questions in steps 1 and 2.

The procedure is not meant to provide a ready set of indicators in advance for any application, but the user is guided and therefore empowered to select its personal set for the respective purpose from a plethora of suggested criteria in literature according to its needs.

Based on our experience in testing the process, the most critical part of the process is the final selection of the criteria set. However, since the procedure is iterative in nature, it is possible to correct previous steps, go through the procedure again and determine an updated set if the user feels that the final set does not meet the requirements. In general, it was found during execution of the step-by-step approach that tabular display (like in Table 3) makes it easier to carry out the proposed procedure. It can be individually adapted by each user to his or her case. Therefore, it is advisable to create a table retracing the different steps and the corresponding key questions and then fill out the table step by step using the procedure.

Notably, many research projects face the challenge of identifying and applying indicators to measure the effectiveness of NbS (Dumitru and Wendling 2021; Sowińska-Świerkosz and García 2021). As elaborated in Section “Existing approaches for NbS evaluation and monitoring”, the existing approaches in literature provide either a holistic but comprehensive set of indicators (Raymond et al. 2017a, 2017b; Dumitru and Wendling 2021) or only specific sets for certain aspects (Liquete et al. 2016; Watkin et al. 2019) or phases of the NbS implementation process (Somarakis et al. 2019; Wendling et al. 2019; Albert et al. 2020). However, a definitive list of assessment indicators that can anticipate all possible cases does not yet exist and probably never will. The particular context (temporal, spatial, sociological, etc.) influences the impacts of NbS, which makes compiling such a list even more difficult. This does not mean that work previously done on assessment of NbS is not helpful; but also to use existing lists of suggested indicators (e.g. Dumitru and Wendling 2021), the user needs guidance to select the appropriate criteria and indicators for his or her individual case in a structured way—so that the selection is comprehensible and replicable. Such guidance has been lacking in the literature and was therefore proposed in this article. The procedure allows suitable and meaningful criteria and indicators to be filtered out from the plethora the ones proposed in the scientific literature in a series of logical steps. Due to its flexibility, it is applicable in different phases of the NbS co-creation process, from the planning to the implementation, with a strong focus on the procedural aspects (i.e.stakeholders). Although these phases may require different assessment tasks and consultancy services, the procedure is suitable for determining the required metrics. At the same time it remains open for adaptation as research and experience on the topic develop further.

For decision-makers, e.g. in city administrations, the procedure can support the planning of future NbS projects by providing tools to collect information on already implemented NbS. In this sense, decision-makers can benefit from targeted information on the performance of NbS by selecting meaningful criteria and indicators for their assessment. However, the selection of the right actors for criteria and indicators identification process is of utmost importance, although this aspect was not focussed very much this paper. Further research could be conducted in this regard.

## Conclusions

This article presented an easy-to-use, and structured procedure to support selecting appropriate criteria and indicators for assessing NbS projects. The procedure is designed to be adaptable to any type of NbS project, especially to the urban context in a co-creative environment, and it tries to structure the complexity and therefore to ease the assessment procedure. It formalizes the identification of appropriate indicators for monitoring and evaluation of all relevant NbS co-creation phases. With the help of the exemplary execution of the procedure in Section “Application”, the applicability could be demonstrated in the first trial in more practical terms.

The exemplary application of the procedure was tested by project partners and found to be easy, logical and quick. The proposed tabular display facilitates the criteria and indicator finding process and provides a solid frame in which the user can approach the right indicators step by step. Further the table with the structured questions can serve as a basis for discussion with stakeholders to consider their needs and views during the criteria and indicator finding process. Nevertheless, further applications and testing are needed for the different types and phases of NbS projects. It is expected that future applications in more real cases will help to further refine and improve the approach proposed in this paper. However, it can be stated that the procedure presented here contains various elements that are advantageous for a successful application:

- Flexibility and openness of the procedure
- Build upon existing indicator lists and data collections
- Consideration of different demands for output monitoring and process evaluation
- Consideration of the needs of different development phases of NbS

## Supplementary Information

Below is the link to the electronic supplementary material.

Supplementary file1 (PDF 17 kb)
