# Supplementary material for: A general procedure to identify indicators for evaluation and monitoring of nature-based solution projects
Source: Ambio. 2022 May 31;51(11):2278–93. doi: 10.1007/s13280-022-01740-0 (PMC9481860; doi:10.1007/s13280-022-01740-0)
Supplement: Supplementary file 1 — Supplementary file1 (PDF 17 kb) [file 13280_2022_1740_MOESM1_ESM.pdf]

Ambio

Electronic Supplementary Material

This supplementary material has not been peer reviewed.

Title: **A general procedure to identify indicators for evaluation and monitoring of Nature-based Solution projects**

Authors: Anne Rödl; Alessandro Arlati

Table S1: Selected criteria and indicators for the different NbS co-creation phases and their results (Arlati et al. 2020)

Phase	Evaluation object	Criteria	Indicator (example)
**Planning/ decision**	Process	- Stakeholder involvement	- Number of respective groups
		- Satisfaction of involved persons	- Perceived satisfaction
		- Involved Experts	- Number of respective experts
		- Speed of agreement	- Time taken for planning<br>- Time taken for decision
	Result	- Suitable to solve the challenges	- Number of challenges addressed by NBS
		- Feasibility	- All parts of the plan clear and technical feasible
		- Fit to the budget	- The budget is not overrun
**Implementation**	Process	- Stakeholder involvement	- Number of respective groups
		- Satisfaction of involved persons	- Perceived satisfaction
		- Time compliance	- Days taken longer than planned
		- Budget compliance	- Money spent over the budget
	Result	- Quality	- Stability of construction<br>- Destruction of the house façade<br>- Share of plants that survived after planting
**Follow-up**	Process	- Status of the façade one year after installation	- Share of surviving plants<br>- Types of surviving plants
	Result	- Improvement of the district	- Increased share of green facades<br>- Knowledge of NBS and marketing of successful projects<br>- Job opportunities for professionals
		- Improvement of biodiversity	- Number of indicator species
